# Structural and biochemical characterization of the M405S variant of *Desulfovibrio vulgaris* formate dehydrogenase

**DOI:** 10.1107/S2053230X24003911

**Published:** 2024-05-01

**Authors:** Guilherme Vilela-Alves, Rita Rebelo Manuel, Neide Pedrosa, Inês A. Cardoso Pereira, Maria João Romão, Cristiano Mota

**Affiliations:** a UCIBIO, Applied Molecular Biosciences Unit, Department of Chemistry, NOVA School of Science and Technology, Universidade NOVA de Lisboa, 2829-516 Caparica, Portugal; b Associate Laboratory i4HB – Institute for Health and Bioeconomy, NOVA School of Science and Technology, Universidade NOVA de Lisboa, 2829-516 Caparica, Portugal; c Instituto de Tecnologia Química e Biológica António Xavier, Universidade NOVA de Lisboa, Avenida da República, 2780-157 Oeiras, Portugal; University of Leipzig, Germany

**Keywords:** CO_2_ reduction, metal-dependent formate dehydrogenases, *Desulfovibrio vulgaris*, Mo/W enzymes, X-ray crystallography, catalysis

## Abstract

Molybdenum- or tungsten-dependent formate dehydrogenases have emerged as significant catalysts for the chemical reduction of CO_2_ to formate, with biotechnological applications envisaged in climate-change mitigation. The role of Met405 in the active site of *Desulfovibrio vulgaris* formate dehydrogenase AB (*Dv*FdhAB) has remained elusive. However, its proximity to the metal site and the conformational change that it undergoes between the resting and active forms suggests a functional role. In this work, the M405S variant was engineered, which allowed the active-site geometry in the absence of methionine S^δ^ interactions with the metal site to be revealed and the role of Met405 in catalysis to be probed. This variant displayed reduced activity in both formate oxidation and CO_2_ reduction, together with an increased sensitivity to oxygen inactivation.

## Abstract 2

A crystallographic and biochemical study of the M405S variant of *D. vulgaris* formate dehydrogenase AB is reported in order to clarify the role of Met405 in catalysis in an enzyme that has applications in climate-change mitigation tools.

## 1. Introduction

The conversion of CO_2_ to value-added products is in high demand as climate-change mitigation measures become increasingly relevant. Nonetheless, these are chemically challenging reactions as CO_2_ is inherently thermodynamically stable, posing extensive challenges to an energy-efficient industrial implementation (Appel *et al.*, 2013 ▸; Calzadiaz-Ramirez & Meyer, 2022 ▸; Aresta *et al.*, 2013 ▸). Metal-dependent formate dehydrogenases (Fdhs) have been tailored by evolution to accomplish this reaction efficiently, selectively and with high activity, leading to an increasing interest in the development of optimized versions of these enzymes as well as of bio-inspired inorganic catalysts (Szczesny *et al.*, 2020 ▸; Lodh & Roy, 2022 ▸; Kanega *et al.*, 2020 ▸).

Molybdenum/tungsten-dependent Fdhs present diverse quaternary structures, allowing them to assume diverse metabolic roles (da Silva *et al.*, 2013 ▸) while interacting with a large assortment of electron acceptors/mediators that are located in membranes or soluble in the cytoplasm and periplasm (Boyington *et al.*, 1997 ▸; Jormakka *et al.*, 2002 ▸; Radon *et al.*, 2020 ▸; Young *et al.*, 2020 ▸; Oliveira *et al.*, 2020 ▸; Raaijmakers *et al.*, 2002 ▸). These enzymes accommodate a wide diversity of β and/or γ subunits harbouring iron–sulfur clusters, haem groups and flavins, but the catalytic subunit (α subunit) is ubiquitously conserved. This subunit contains the molybdenum/tungsten active site and one [4Fe–4S] cluster (for electron shuttling) and is responsible for the reversible conversion of formate to CO_2_ with a substantially higher turnover than its metal-independent counterparts (Maia *et al.*, 2015 ▸; Nielsen *et al.*, 2019 ▸).

In the active site, the Mo/W atom coordinates two dithiolenes from two molybdopterin guanine dinucleotides (MGDs), one terminal sulfido ligand (—SH/=S) and a (seleno)cysteine residue from the polypeptide chain in a distorted trigonal prismatic geometry (Romão, 2009 ▸; Hille *et al.*, 2014 ▸; Grimaldi *et al.*, 2013 ▸). The two MGD ligands are structurally equivalent but have different roles. MGD1 (the proximal MGD) is involved in electron transfer from the molybdenum/tungsten centre to the first [4Fe–4S] cluster (Oliveira *et al.*, 2020 ▸; Raaijmakers *et al.*, 2002 ▸; Supplementary Fig. S1). MGD2 (the distal MGD) is believed to play a role in modulation of the molybdenum/tungsten redox potential (Oliveira *et al.*, 2020 ▸). In the second coordination sphere of the metal, two conserved residues are catalytically relevant: a histidine thought to play a role in proton transfer (Oliveira *et al.*, 2020 ▸) and an arginine involved in substrate orientation and stabilization of putative catalytic intermediates (Oliveira *et al.*, 2020 ▸; Siegbahn, 2022 ▸).

In the particular case of the tungsten FdhAB from *Desulfovibrio vulgaris* (*Dv*FdhAB), a selenocysteine coordinates the tungsten ion and the enzyme displays a simple heterodimeric structure: a catalytic α subunit (harbouring the tungsten active site and one [4Fe–4S] centre) and a β subunit (with three [4Fe–4S] clusters) involved in electron transfer to/from electron acceptors/donors (Oliveira *et al.*, 2020 ▸).

In spite of several studies, doubts persist regarding the catalytic mechanism, particularly in relation to the dissociation of the (seleno)cysteine during catalysis (Raaijmakers & Romão, 2006 ▸; Schrapers *et al.*, 2015 ▸; Oliveira *et al.*, 2022 ▸) and/or the transfer of a formal hydride (H^+^ + 2e^−^) between the metal site and the substrate (Schrapers *et al.*, 2015 ▸; Niks & Hille, 2019 ▸; Siegbahn, 2022 ▸).

In a recent study of *Dv*FdhAB, a disulfide redox switch was shown to be crucial in an allosteric mechanism for enzyme activation (yielding maximum activity) or inactivation (resulting in protection against O_2_ damage) (Oliveira *et al.*, 2024 ▸). When the disulfide bond is reduced, the highest catalytic activity for CO_2_ reduction is achieved. On the other hand, in the presence of the disulfide bond the *k*_cat_ decreases by 90% and the *K*_m_ increases. The allosteric mechanism involves large conformational changes, namely of the side chain of Met405, a residue located near the active site that is fully conserved in the subclass of Fdhs that contain the recently reported allo­steric redox switch for protection against oxygen-induced damage. The M405A mutation virtually abolished the catalytic activity (Oliveira *et al.*, 2024 ▸) and its structure revealed a significant distortion of the active site, particularly the protein backbone near SeCys192 (U192), preventing modelling of the side chain of U192. The mutation proved to be too severe, preventing clarification of the possible catalytic role of Met405 and suggesting a major structural role for this residue.

Here, we report the crystal structure of the M405S variant of *Dv*FdhAB and its kinetic characterization. The hexacoordinated tungsten active site could now be fully modelled and allowed the prominent role of Met405 in the geometry of the metal site to be probed.

## 2. Materials and methods

### 2.1. Expression, purification and kinetic assays of *D. vulgaris* FdhAB

*Dv*FdhAB M405S was expressed and affinity-purified from *D. vulgaris* Hildenborough as described in Oliveira *et al.* (2020 ▸) (Table 1 ▸). Affinity chromatography was performed aerobically and anaerobically using Strep-TactinXT 4Flow resin (IBA Lifesciences), eluting the protein with 100 m*M* Tris–HCl buffer containing 150 m*M* NaCl and 50 m*M* biotin. Anaerobic purification was performed inside a Coy anaerobic chamber (2% H_2_/98% N_2_) using the same method as aerobic purification. The protein concentration was determined based on ɛ_410 nm_ = 43.45 m*M* ^−1^ cm^−1^. After protein purification, kinetic assays were performed as reported in Oliveira *et al.* (2020 ▸) with DTT pretreatment and a final *Dv*FdhAB M405S concentration of 14 n*M* using a UV-1800 Shimadzu spectrophotometer inside a Coy anaerobic chamber (2% H_2_/98% N_2_).

### 2.2. Crystallization, data collection, structure solution and refinement

To obtain the crystallization conditions for the M405S variant, we started from the wild-type (WT) crystallization conditions reported in Oliveira *et al.* (2020 ▸) and performed microseeding. The conditions were optimized by varying the PEG 3350 concentration and by microseed dilution.

*Dv*FdhAB M405S [15 mg ml^−1^ in 20 m*M* Tris–HCl pH 7.6, 10%(*v*/*v*) glycerol, 10 m*M* NaNO_3_ buffer] was crystallized at 20°C using the hanging-drop vapour-diffusion method in 24-well plates (24-well XRL plates from Molecular Dimensions) with drops containing precipitant solution consisting of 32%(*w*/*v*) PEG 3350, 0.1 *M* Tris–HCl pH 8.0, 1 *M* LiCl (Oliveira *et al.*, 2020 ▸) and a 1:500 dilution from a stock of microseeds of *Dv*FdhAB WT with a 1:1:0.2 ratio of protein:precipitant:microseeds (Table 2 ▸).

Crystals appeared within 24 h, grew for 72 h and were flash-cooled in liquid nitrogen using the harvesting buffer supplemented with 20%(*v*/*v*) glycerol as a cryoprotectant.

X-ray diffraction experiments were performed on beamline ID30B (McCarthy *et al.*, 2018 ▸) at the ESRF synchrotron, Grenoble, France. The crystals were cryocooled at 100 K and data processing was performed using *XDS* (Kabsch, 2010 ▸) and *STARANISO* (Vonrhein *et al.*, 2018 ▸). As the data showed anisotropy, *STARANISO* was used to improve the overall quality of the final electron-density maps. Molecular replacement with *Phaser* (McCoy *et al.*, 2007 ▸) from the *CCP*4 suite (Agirre *et al.*, 2023 ▸) was used to solve the structure using the oxidized WT *Dv*FdhAB structure (PDB entry 6sdr) as a search model. The model was iteratively refined by cycles of automatic restrained refinement with *REFMAC*5 (Murshudov *et al.*, 2011 ▸) and manual model building with *Coot* (Emsley *et al.*, 2010 ▸). The model was rebuilt using the online *PDB-REDO* server (Joosten *et al.*, 2009 ▸) and publication images were generated with *PyMOL* (Schrödinger). Data-collection and processing statistics and model-refinement statistics are presented in Tables 3 ▸ and 4 ▸, respectively.

## 3. Results and discussion

### 3.1. Biochemical characterization of *Dv*FdhAB M405S

**Figure 1:**
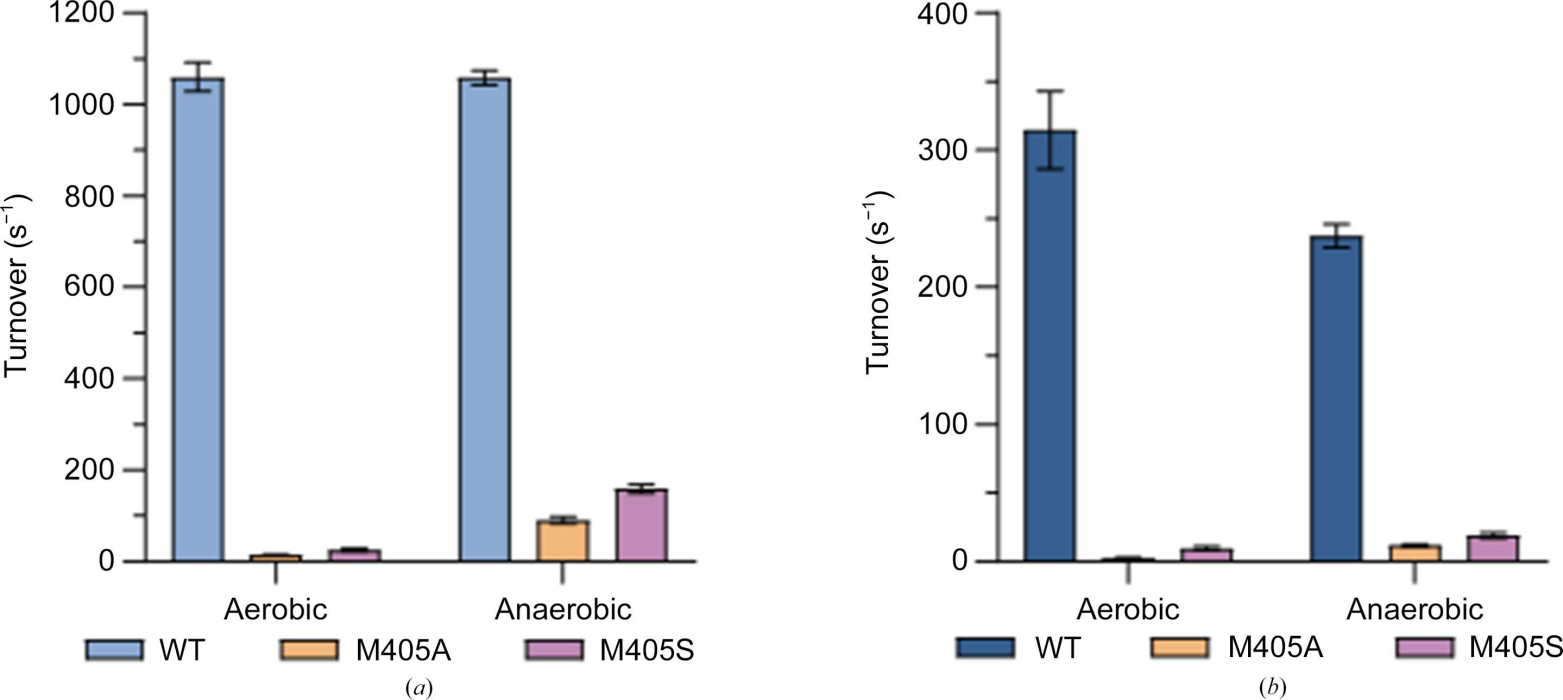
Turnover rates of WT *Dv*FdhAB and its M405A and M405S variants from aerobic and anaerobic purification. (*a*) Turnover rates for formate oxidation by WT *Dv*FdhAB (light blue) and its M405A (orange) and M405S (purple) variants. (*b*) Turnover rates for CO_2_ reduction by WT *Dv*FdhAB (dark blue) and its M405A (orange) and M405S (purple) variants. Data are presented as mean values ± s.d. (*n* = 3 technical replicates of the assay).

The *Dv*FdhAB M405S variant presents an eightfold lower activity for formate oxidation and an 18-fold lower activity for CO_2_ reduction compared with the WT enzyme (Table 5 ▸). A strong loss of activity was previously reported for the M405A variant, where the activities were 16-fold and eightfold lower than those of WT *Dv*FdhAB for formate oxidation and CO_2_ reduction, respectively (Table 5 ▸; Oliveira *et al.*, 2024 ▸). The *K*_m_ of the M405S variant for formate was twice that of the WT and the M405A variant (Table 5 ▸). On the other hand, the *K*_m_ for CO_2_ was equally high for both variants and was two orders of magnitude higher than that of the WT (Oliveira *et al.*, 2024 ▸). Although the difference in the kinetic parameters of both variants relative to the WT was not the same, the efficiencies (*k*_cat_/*K*_m_) for formate oxidation and CO_2_ reduction were similar (Table 5 ▸). The activities of both variants increased slightly when the proteins were purified anaerobically (Figs. 1 ▸ *a* and 1 ▸ *b*). This reveals that the variants are more sensitive to oxygen than WT *Dv*FdhAB, for which the activity after anaerobic purification did not increase (Oliveira *et al.*, 2024 ▸; Figs. 1 ▸ *a* and 1 ▸ *b*). The activity profile of the M405S variant is similar to that observed for the M405A variant (Oliveira *et al.*, 2024 ▸) and the results confirm and reinforce that Met405 is essential for the activity and substrate affinity of *Dv*FdhAB. Furthermore, mutation of this residue results in an increased sensitivity to oxygen.

### 3.2. Structure of *Dv*FdhAB M405S

**Figure 2:**
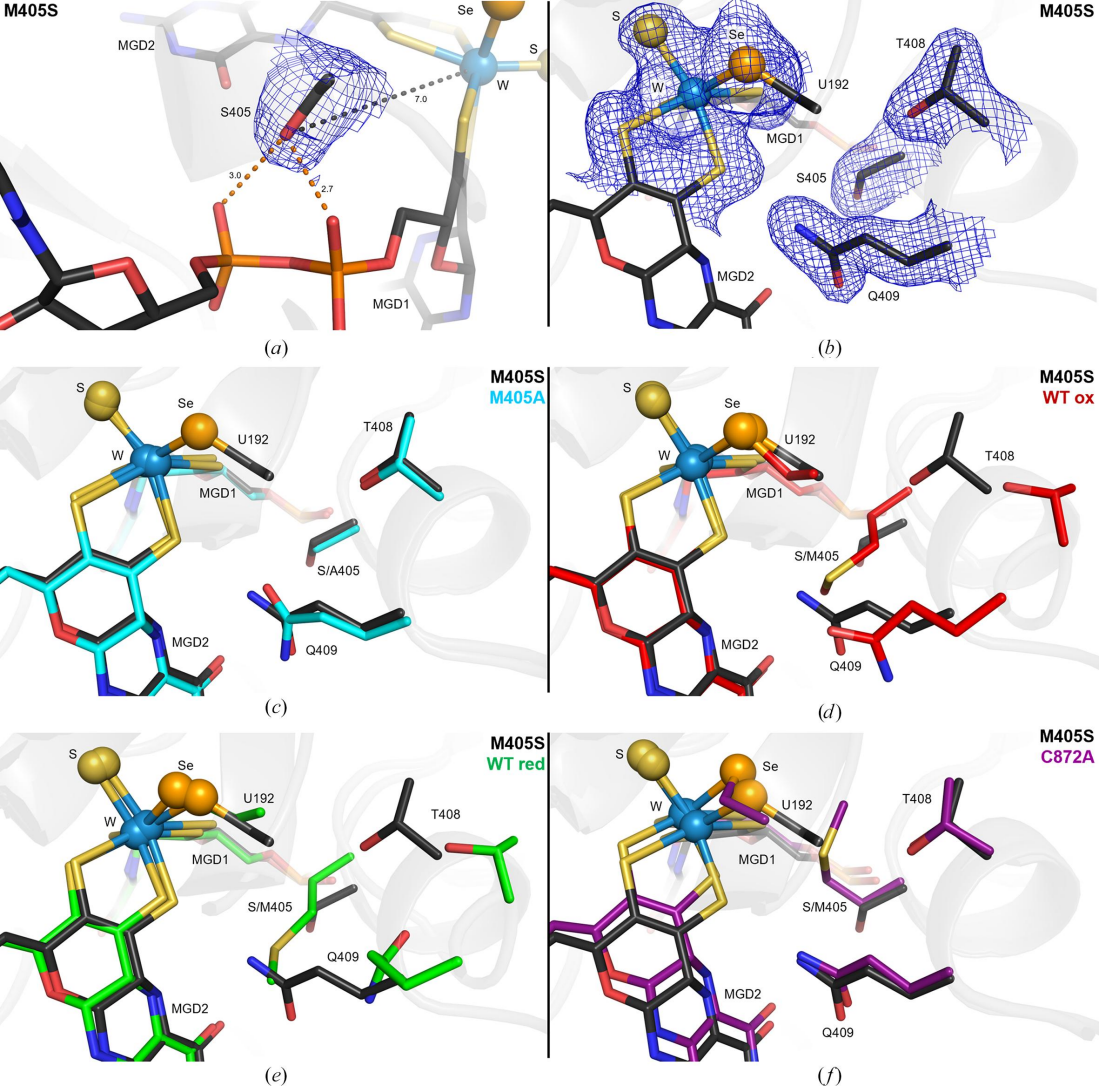
Effect of the M405S mutation on the structure of *Dv*FdhAB. In all representations, the tungsten active site is shown as sticks; the tungsten ion, sulfido group and the U192 Se atom are shown as spheres (light blue, yellow and orange, respectively). (*a*) The M405S mutation is shown as sticks in the structure of the M405S variant (black). 2*F*_o_ − *F*_c_ electron-density maps at 1σ are shown as a blue mesh for the side chain of Ser405. The Ser405 O^γ^–W distance and the hydrogen bonds established to O2α and O2β from the phosphate groups of MGD1 are shown as black and orange dashed lines, respectively. Distances are shown in Å. (*b*) The M405S variant (black) in a different orientation. Ser405 and the residues with greater conformational changes (U192, Thr408 and Gln409) are shown as sticks and their respective 2*F*_o_ − *F*_c_ electron-density maps at 1σ are shown as a blue mesh. (*c*) A superposition of the M405S (black) and M405A (PDB entry 8cm7; cyan) variants is shown. The mutated residue (Met405) and the residues with greater conformational rearrangement in the M405S variant (U192, Thr408 and Gln409) are shown as sticks. In the M405A variant the U912 side chain could not be modelled and is absent in the deposited structure. (*d*) A superposition of the M405S variant (black) and the oxidized WT (red; PDB entry 6sdr) is shown in the same orientation as in (*c*). (*e*) A superposition of the M405S variant (black) and the reduced WT (green; PDB entry 6sdv) is shown in the same orientation as in (*c*). (*f*) A superposition of the M405S (black) and C872A (violet; PDB entry 8cm6) variants is shown in the same orientation as in (*c*).

*Dv*FdhAB M405S yielded crystals that belonged to space group *P*2_1_2_1_2_1_ and diffracted to 2.0 Å resolution (Table 3 ▸). The diffraction pattern showed anisotropy and the data were processed using *STARANISO* to improve the statistics and maps (Vonrhein *et al.*, 2018 ▸). Considering an ellipsoid cutoff, the statistics showed a completeness of 93.02% and 49.47% for the overall data set and the highest resolution shell, respectively, an overall CC_1/2_ of 0.996 for the overall data set and 0.562 for the highest resolution shell, and a 〈*I*/σ(*I*)〉 of 1.5 for the last shell. The model was refined with good geometry indicators (0.17% Ramachandran outliers and a *MolProbity* score of 1.60 Å) with *R*-factor and *R*_free_ values of 0.212 and 0.253, respectively (Table 4 ▸). As expected, the overall structure of the M405S variant is very similar to the WT and to M405A variant structures. Superimposition of the M405S variant with oxidized WT *Dv*FdhAB (PDB entry 6sdr) yields an r.m.s.d. of 0.37 Å for 1177 C^α^ atoms, superimposition with the reduced WT enzyme (PDB entry 6sdv) yields an r.m.s.d. of 0.40 Å for 1177 C^α^ atoms and superimposition with the M405A variant (PDB entry 8cm7) yields an r.m.s.d. of 0.28 Å for 1170 C^α^ atoms. However, the mutation induced structural changes in the protein backbone near the tungsten active site and its ligands (Fig. 2 ▸). In the previously reported structure of the M405A variant, the structural changes in this region were unclear due to very poor electron density; the selenocysteine side chain could not be modelled and was omitted from the deposited structure (PDB entry 8cm7).

### 3.3. Structure comparison of *Dv*FdhAB M405S with other variants and the wild type

In the new variant the electron density for the Ser405 side chain is well defined and its O^γ^ atom is stabilized by hydrogen bonds to O2α and O2β of two phosphate groups of MGD1 (Fig. 2 ▸ *a*). This interaction, which is not present in M405A, stabilizes this region and may provide an explanation for the enhanced quality of the crystals of the M405S variant and for the lack of disorder at the tungsten active site (Fig. 2 ▸ *b*).

The mutation of Met405 to serine (or alanine) led the Gln409 side chain to occupy the space left vacant by the absence of the Met405 side chain, resulting in a backbone rearrangement that pulls Thr408 4.3 Å away from its original position and causes it to interact with the protein backbone near U192 (Figs. 2*c*, 2 ▸ *d* and 2 ▸ *e*). Gln409 and Thr408 are superposed in M405S and C872A variants (Fig. 2*f* ▸).

**Figure 3:**
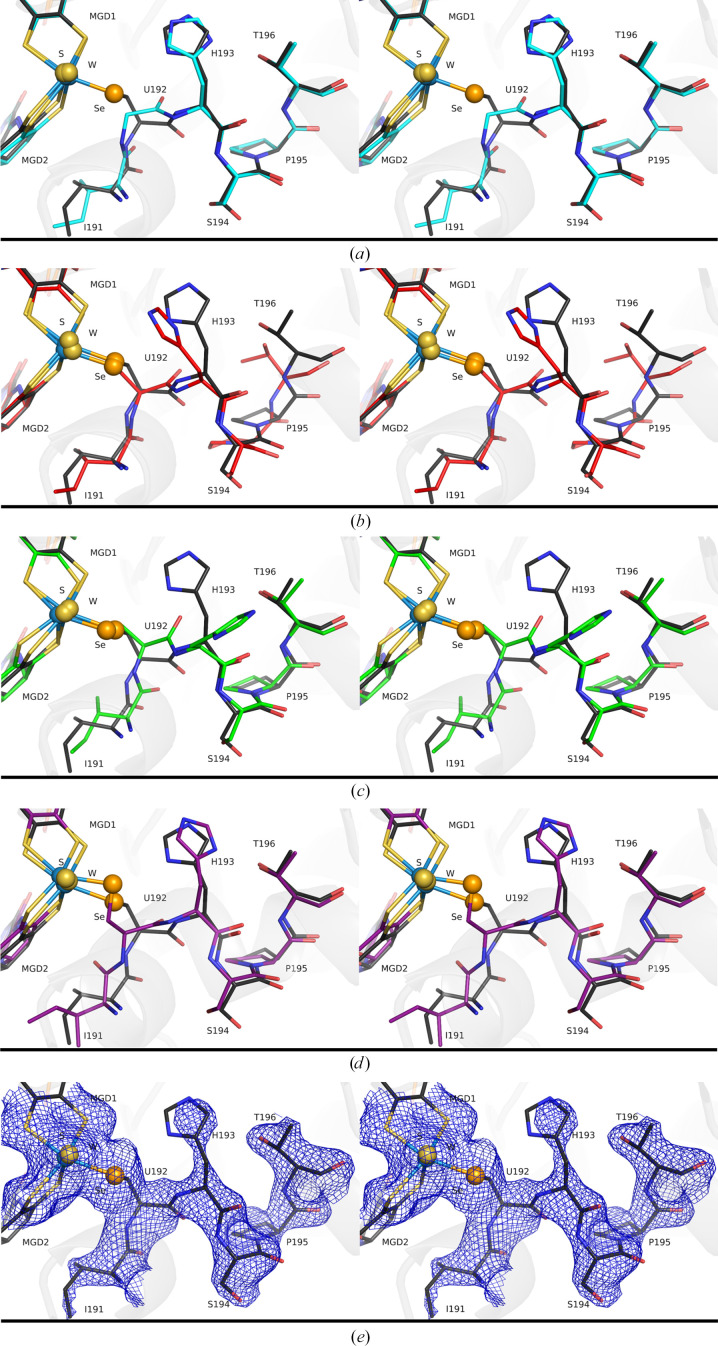
Effect of the absence of the Met405 side chain on the conformation of the helix region Ile191–Thr196 in *Dv*FdhAB. In all stereo representations, the tungsten active site and the helix portion Ile191–Thr196 are shown as sticks; the tungsten ion, sulfido group and U192 Se atom are shown as spheres (light blue, yellow and orange, respectively). (*a*) Superposition of the M405S (black) and M405A (PDB entry 8cm7; cyan) variants. (*b*) Superposition of the M405S variant (black) and the oxidized WT (PDB entry 6sdr; red). (*c*) Superposition of the M405S variant (black) and the formate-reduced WT (green; PDB entry 6sdv). (*d*) Superposition of the M405S (black) and C872A (violet; PDB entry 8cm6) variants. (*e*) The M405S (black) variant. The 2*F*_o_ −  *F*_c_ electron-density maps at 1σ for the helix region Ile191–Thr196 and for the tungsten active site are shown as a blue mesh.

The helix portion Ile191–Thr196, which is affected by the positioning of Thr408, was modelled in a new conformation (similar to the M405A variant; Fig. 3 ▸) that was not previously seen in the WT (oxidized or reduced) or the recently reported activated C872A variant (PDB entry 8cm6), which mimics the physiological active state of the enzyme (Oliveira *et al.*, 2024 ▸). This helix segment was modelled in a conformation closer to the oxidized WT form (Ile191–Ser194), moving closer to the reduced WT form and the activated C872A form from Pro195 onwards and becoming completely superposable as early as Thr196 (Fig. 3 ▸). Additionally, the catalytically essential residue His193 displays a new conformation that is closer to that in the oxidized WT enzyme, but is intermediate between the oxidized and reduced states. Therefore, this new conformation, which is associated with increased backbone flexibility, is likely to interfere with the catalytic role of His193, possibly accounting for some of the loss of activity of the M405S variant. Nonetheless, using the current data we cannot determine its exact impact on the catalytic activity.

**Figure 4:**
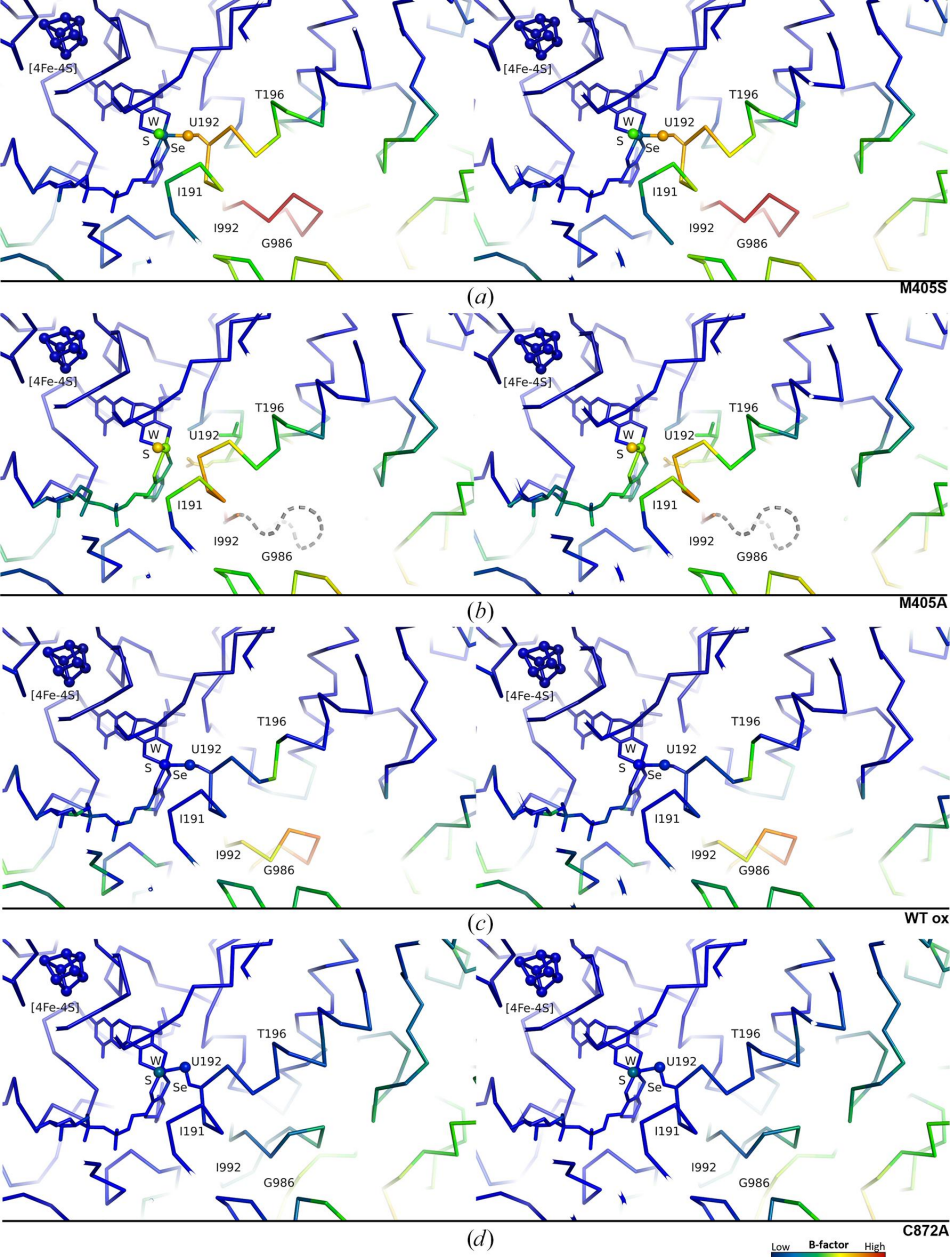
Effect of the absence of the Met405 side chain on the *B* factors of the C^α^ atoms of Ile191–Thr196 of *Dv*FdhAB. In all segments, the tungsten active site and the proximal [4Fe–4S] cluster are shown, in stereo representation, as sticks and spheres, the peptide chain is shown in ribbon representation and the *B*-factor colour scale is shown. *B* factors were normalized for each structure independently. (*a*) The M405S variant. (*b*) The M405A variant (PDB entry 8cm7). The loop Gly896–Ile992, which was not modelled for this data set, is represented as a grey dashed line. (*c*) The oxidized WT (PDB entry 6sdr). (*d*) The C872A variant (PDB entry 8cm6).

As in the M405A variant, the *B* factors in this region (Ile191–Thr196) of the M405S variant are very high, indicating flexibility of this helix segment arising from the absence of the large residue (Fig. 4 ▸). Only loop Gly986–Ile992, which is located at the entrance to the substrate channel, has higher *B* factors than Ile191–Thr196 within the protein core (Fig. 4 ▸ *a*), as observed in other Fdh structures (Vilela-Alves *et al.*, 2023 ▸).

### 3.4. The tungsten-site geometry

**Figure 5:**
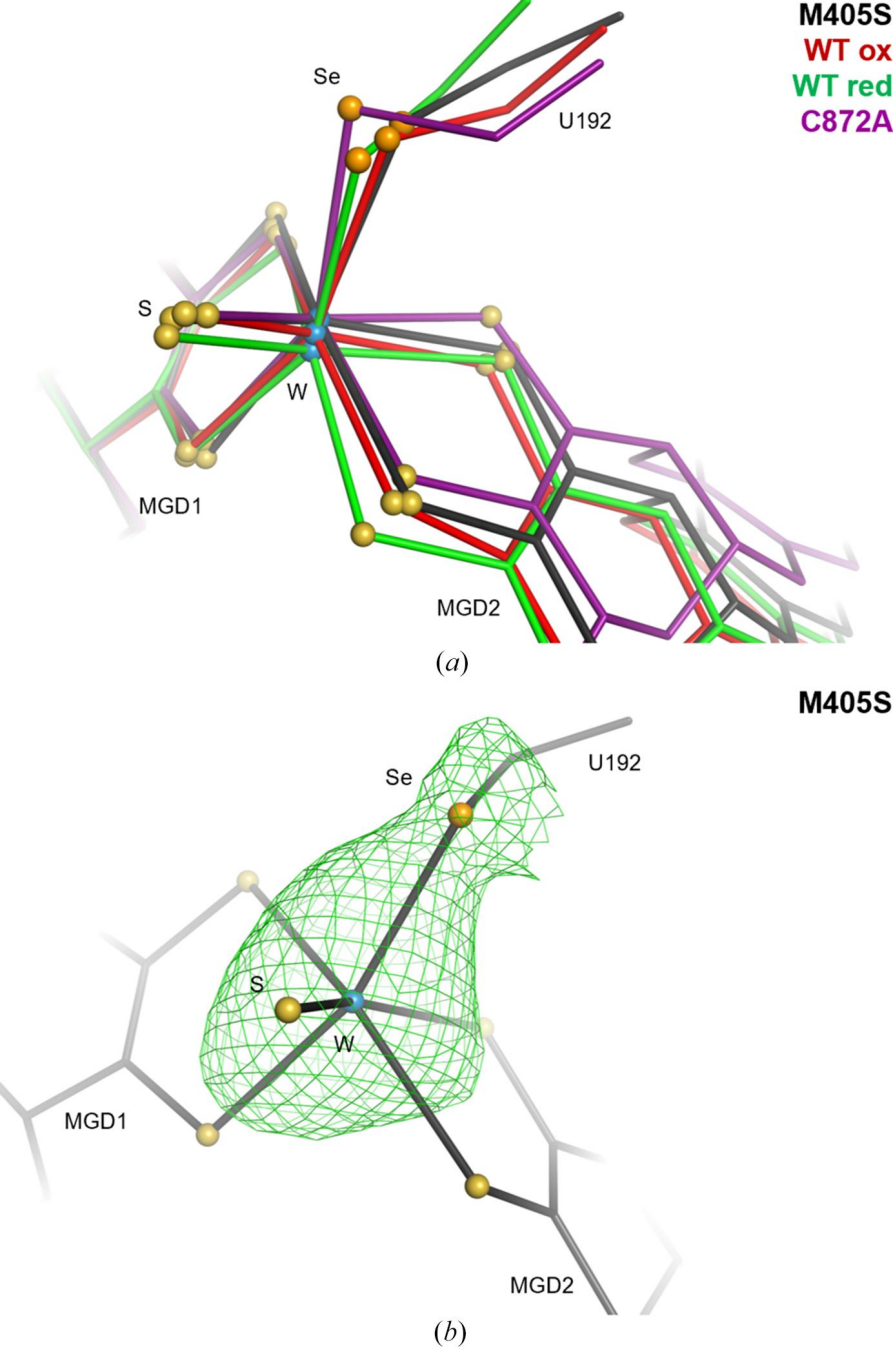
Effect of the absence of the Met405 side chain on the geometry of the tungsten active site in *Dv*FdhAB. In all segments, the tungsten active site is shown as sticks and the tungsten ion, the S atoms (sulfido ligand and MGDs dithiolene groups) and the U192 Se atom are shown as spheres (light blue, yellow and orange, respectively). (*a*) A superposition of the M405S variant (black), the oxidized WT (red; PDB entry 6sdr), the formate-reduced WT (green; PDB entry 6sdv) and the C872A variant (violet; PDB entry 8cm6) is shown. The structures were aligned using MGD1, as this cofactor shows the minimal conformational variability between the four structures. (*b*) The M405S variant (black) is shown. The anomalous map peaks at 3σ are shown as a green mesh for the W and Se atoms.

**Figure 6:**
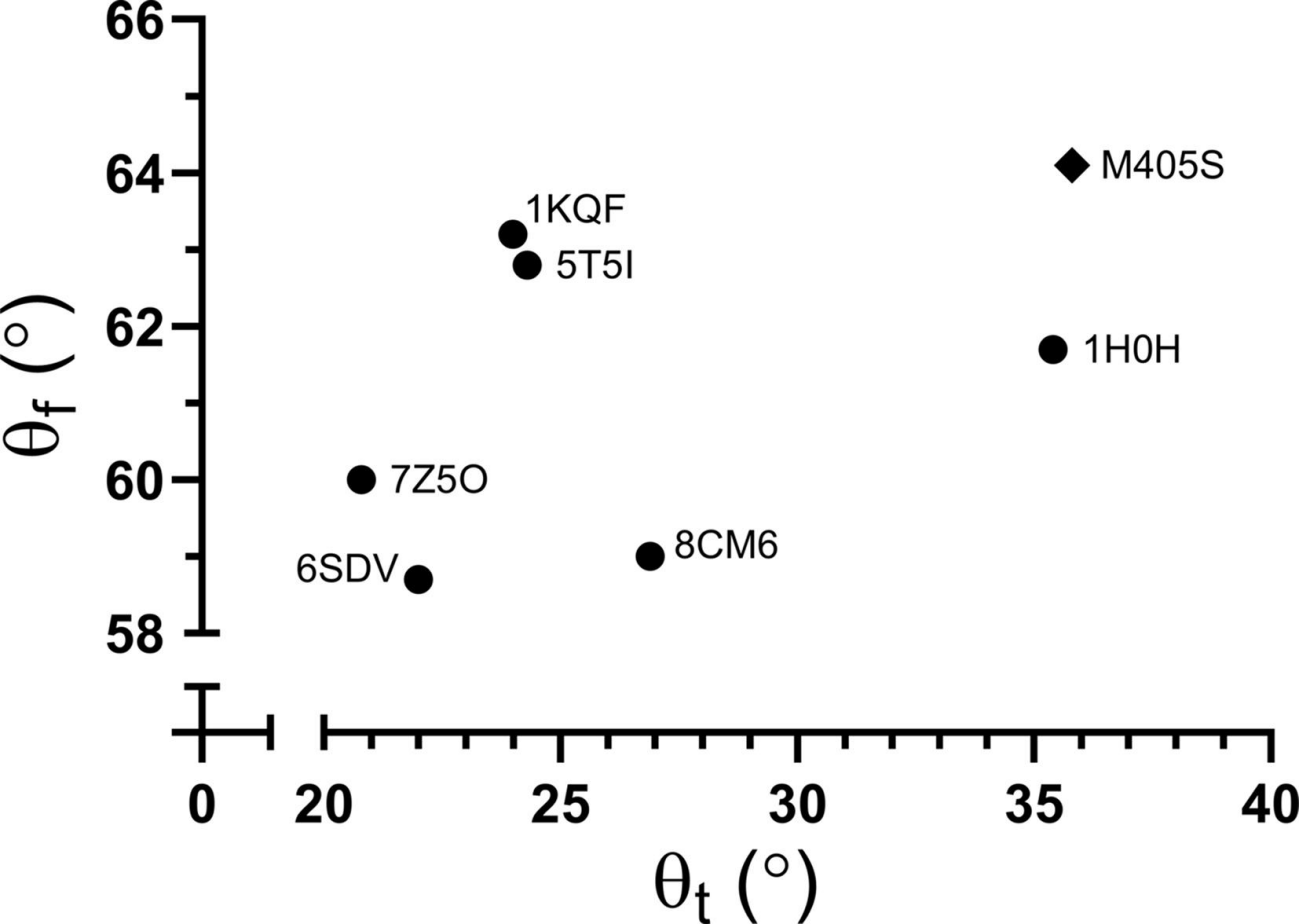
Active-site twisting (θ_t_) and folding (θ_f_) angles of the pterin planes for different forms and/or variants of Fdh (structures with resolutions above 2 Å). Structures are identified by their respective PDB codes: 6sdv, formate-reduced *Dv*FdhAB; 7z5o, dithionite-reduced *Dv*FdhAB; 8cm6, *Dv*FdhAB C872A_anox; 1kqf, *E. coli* FdhN; 1h0h, *Desulfovibrio gigas* FdhAB; 5t5i, *Methanobacterium wolfeii* FMFD.

In the structure of the M405S variant (in contrast to that of the M405A variant) the selenocysteine side chain could be modelled and the selenium location was clearly identified by the anomalous difference map (up to 4σ; Fig. 5 ▸ *b*), enabling a direct comparison of the tungsten active site (WT versus M405S variant; Fig. 5 ▸ *a*). The active site of the M405S variant is also significantly different from that in the C872A variant (equivalent to the active state of the enzyme), suggesting that the low activity displayed by the M405S variant is likely to be due to its altered active site. To better characterize the active-site geometry, the twisting and folding angles of the MGD dithiolenes were calculated as defined by Liu *et al.* (2022 ▸). The twisting angle is the angle obtained from the scalar product of the two vectors connecting the two S atoms within the same dithiolene ligand and the folding angle is defined as 180° − θ, where θ is the angle formed by the two vectors connecting the metal ion and the midpoint between the S atoms from the same dithiolene ligand. The twisting angle is intended to assess the extent to which the dithiolene S atoms lie in the same plane, whereas the folding angle gauges how much the metal ion is pulled out of the plane of the four S atoms (Liu *et al.*, 2022 ▸). Comparison of the twisting and folding angles of the active-site coordination is presented in Fig. 6 ▸. Ultimately, the substantial disparity between the tungsten active-site geometry of the M405S variant and the highly active form of the C872A variant (PBD entry 8cm6), as shown by distinct twisting and folding angles of 35.8° and 26.9° and of 64.1° and 59.0°, respectively (Fig. 6 ▸), is probably correlated with the diminished reactivity of this variant. Adding to this, the metal site in the M405S variant presents higher *B* factors, suggesting instability (flexibility or metal depletion), which will also affect the activity.

## 4. Conclusion

The crystal structure of the M405S variant of *Dv*FdhAB allowed us to unequivocally model the complete catalytic site of an inactive form of the enzyme. The replacement of a large hydrophobic side chain by a polar and short residue, stabilized by hydrogen bonds, allowed the tungsten coordination sphere to be retained, resulting in well defined electron density to model the tungsten ligand, U192, with a clear position for the Se atom. Met405 was previously assigned to play a key structural role, but only in the present M405S variant could we confirm the significant rearrangement of the Ile191–Thr196 helical region (particularly the mechanistically relevant residues U192 and His193) and the consequent distortion of the metal coordination geometry. These observed structural changes complemented by the reported loss of activity confirm previously published results. To elucidate the putative catalytic role of Met405, further mutagenesis experiments are being planned.

## Supplementary Material

PDB reference: M405S variant of *Desulfovibrio vulgaris* formate dehydrogenase AB, 8rcg

Supplementary Figure S1. DOI: 10.1107/S2053230X24003911/no5205sup1.pdf

## Figures and Tables

**Table 1:** Macromolecule-production information

Source organism	*D. vulgaris* Hildenborough
DNA source	Mutated native DNA sequence
Expression vector	pMO9075
Plasmid construction method	Sequence- and ligation-independent cloning (SLIC) and site-directed mutagenesis
Forward primer (cloning)	
FdhA	5′-GCAGTCCCAGGAGGTACCATATGACAGTCACACGCAGACATTTCC-3′
FdhB	5′-CGCCTTCTTGACGAGTTCTTCTGAGGAGGTAGGTCATGGGAAAGATGTTCTTC-3′
Reverse primer (cloning)	
FdhA	5′-GAAACGATCCTCATCCCTATTTTTCGAACTGCGGGTGGCTCCAGACCTTGCGGACGTCCACCATG-3′
FdhB	5′-CTTCATGATCGGGTCTTTTCGTTCAGGCACGTGGCCGGAACAA-3′
Expression host	*D. vulgaris* Hildenborough Δ*fdhAB* deletion strain
Complete amino-acid sequence of the protein produced	
FdhA	MTVTRRHFLKLSAGAAVAGAFTGLGLSLAPTVARAELQKLQWAKQTTSICCYCAVGCGLIVHTAKDGQGRAVNVEGDPDHPINEGSLCPKGASIFQLGENDQRGTQPLYRAPFSDTWKPVTWDFALTEIAKRIKKTRDASFTEKNAAGDLVNRTEAIASFGSAAMDNEECWAYGNILRSLGLVYIEHQARIUHSPTVPALAESFGRGAMTNHWNDLANSDCILIMGSNAAENHPIAFKWVLRAKDKGATLIHVDPRFTRTSARCDVYAPIRSGADIPFLGGLIKYILDNKLYFTDYVREYTNASLIVGEKFSFKDGLFSGYDAANKKYDKSMWAFELDANGVPKRDPALKHPRCVINLLKKHYERYNLDKVAAITGTSKEQLQQVYKAYAATGKPDKAGTIMYASGWTQHSVGVQNIRAMAMIQLLLGNIGVAGGGVNALRGESNVQGSTDQGLLAHIWPGYNPVPNSKAATLELYNAATPQSKDPMSVNWWQNRPKYVASYLKALYPDEEPAAAYDYLPRIDAGRKLTDYFWLNIFEKMDKGEFKGLFAWGMNPACGGANANKNRKAMGKLEWLVNVNLFENETSSFWKGPGMNPAEIGTEVFFLPCCVSIEKEGSVANSGRWMQWRYRGPKPYAETKPDGDIMLDMFKKVRELYAKEGGAYPAPIAKLNIADWEEHNEFSPTKVAKLMNGYFLKDTEVGGKQFKKGQQVPSFAFLTADGSTCSGNWLHAGSFTDAGNLMARRDKTQTPEQARIGLFPNWSFCWPVNRRILYNRASVDKTGKPWNPAKAVIEWKDGKWVGDVVDGGGDPGTKHPFIMQTHGFGALYGPGREEGPFPEHYEPLECPVSKNPFSKQLHNPVAFQIEGEKKAVCDPRYPFIGTTYRVTEHWQTGLMTRRCAWLVEAEPQIFCEISKELAKLRGIGNGDTVKVSSLRGALEAVAIVTERIRPFKIEGVDVHMVGLPWHYGWMVPKNGGDTANLLTPSAGDPNTGIPETKAFMVDVRKVWSHPQFEK
FdhB	MGKMFFVDLSRCTACRGCQIACKQWKNLPAEETRNTGSHQNPPDLSYVTLKTVRFTEKSRKGPGIDWLFFPEQCRHCVEPPCKGQADVDLEGAVVKDETTGAVLFTELTAKVDGESVRSACPYDIPRIDPVTKRLSKCDMCNDRVQNGLLPACVKTCPTGTMNFGDEQEMLALAEKRLAEVKKTYPGAVLGDPNDVRVVYLFTRDPKDFYEHAVA

**Table 2:** Summary of crystallization conditions

Method	Vapour diffusion, hanging drop
Plate type	24-well XRL plate (Molecular Dimensions)
Temperature (°C)	20
Protein concentration (mg ml^−1^)	15
Composition of the protein solution	20 m*M* Tris–HCl pH 7.6, 10%(*v*/*v*) glycerol, 10 m*M* NaNO_3_
Composition of the reservoir solution	32%(*w*/*v*) PEG 3350, 0.1 *M* Tris–HCl pH 8.0, 1 *M* LiCl
Volume and ratio of drop	2.2 µl of a 1:1:0.2 µl ratio of protein:precipitant:microseed dilution
Volume of reservoir (µl)	500
Composition of the cryoprotectant	20%(*v*/*v*) glycerol, 32%(*w*/*v*) PEG 3350, 0.1 *M* Tris–HCl pH 8.0, 1 *M* LiCl
Drop setting	Manual
Seeding	Yes, 1:500 dilution of stock WT FdhAB seeds

**Table 3:** Data-collection and processing statistics Values in parentheses are for the outer shell.

	*Dv*FdhAB M405S	*Dv*FdhAB M405S (*STARANISO*)
PDB code		8rcg
Diffraction source	ID30B, ESRF	ID30B, ESRF
Wavelength (Å)	0.8731	0.8731
Temperature (K)	100	100
Crystal-to-detector distance (mm)	231.55	231.55
Rotation range per image (°)	0.05	0.05
Total rotation range (°)	360	360
Space group	*P*2_1_2_1_2_1_	*P*2_1_2_1_2_1_
*a*, *b*, *c* (Å)	64.34, 127.23, 148.27	64.34, 127.23, 148.27
Mosaicity (°)	0.03	0.03
Resolution range (Å)	48.59–2.00 (2.11–2.00)	48.59–2.00 (2.12–2.00)
Total No. of reflections	377283 (60741)	329055 (19634)
No. of unique reflections	83264 (13159)	71441 (3573)
Completeness (%)	99.00 (98.30)	93.02 (49.47)
Multiplicity	4.5 (4.6)	4.6 (5.5)
〈*I*/σ(*I*)〉	7.1 (1.0)	7.4 (1.5)
*R*_meas_	0.150 (1.572)	0.144 (1.305)
CC_1/2_	0.996 (0.375)	0.996 (0.562)

**Table 4:** Refinement statistics Values in parentheses are for the outer shell.

	*Dv*FdhAB M405S (*STARANISO*)
PDB code	8rcg
Resolution range (Å)	48.59–2.00 (2.06–2.00)
Final *R*_cryst_	0.212 (0.333)
Final *R*_free_	0.253 (0.402)
No. of non-H atoms	
Total	9610
Protein	9197
Ligand	167
Ion	2
Water	244
R.m.s. deviations	
Bond lengths (Å)	0.005
Angles (°)	1.313
Average *B* factors (Å^2^)	
Overall	40.17
Protein	43.00
Ligand	36.77
Ion	47.67
Water	34.58
Ramachandran plot	
Most favoured (%)	96.15
Outliers (%)	0.17
*MolProbity* score	1.60
Clashscore	5.42

**Table 5:** Kinetic parameters of WT *Dv*FdhAB and its M405A and M405S variants

		Formate oxidation			CO_2_ reduction			
Purification conditions	Variant	*k*_cat_ (s^−1^)	*K*_m_ (µ*M*)	*k*_cat_/*K*_m_ (s^−1^ m*M* ^−1^)	*k*_cat_ (s^−1^)	*K*_m_ (µ*M*)	*k*_cat_/*K*_m_ (s^−1^ m*M* ^−1^)	Reference
Aerobic	WT	1310 ± 50	17 ± 3	77515	344 ± 41	324 ± 54	1090	Oliveira *et al.* (2020 ▸)
Anaerobic	M405A	82 ± 2	18 ± 2	4603	43 ± 3	12253 ± 1942	4	Oliveira *et al.* (2024 ▸)
Anaerobic	M405S	159 ± 11	40 ± 4	3975	19 ± 2	17753 ± 4225	1	This work
