# Supplementary material for: Structural and biochemical characterization of the M405S variant of Desulfovibrio vulgaris formate dehydrogenase
Source: Acta Crystallogr F Struct Biol Commun. 2024 May 1;80(Pt 5):98–106. doi: 10.1107/S2053230X24003911 (PMC11134731; doi:10.1107/S2053230X24003911)
Supplement: Supplementary file 1 [file f-80-00098-sup1.pdf]

STRUCTURAL BIOLOGY COMMUNICATIONS

**Volume 80 (2024)**

**Supporting information for article:**

## Structural and biochemical characterization of the M405S variant of *Desulfovibrio vulgaris* formate dehydrogenase

**Guilherme Vilela-Alves, Rita Rebelo Manuel, Neide Pedrosa, Inês A. Cardoso Pereira, Maria João Romão and Cristiano Mota**

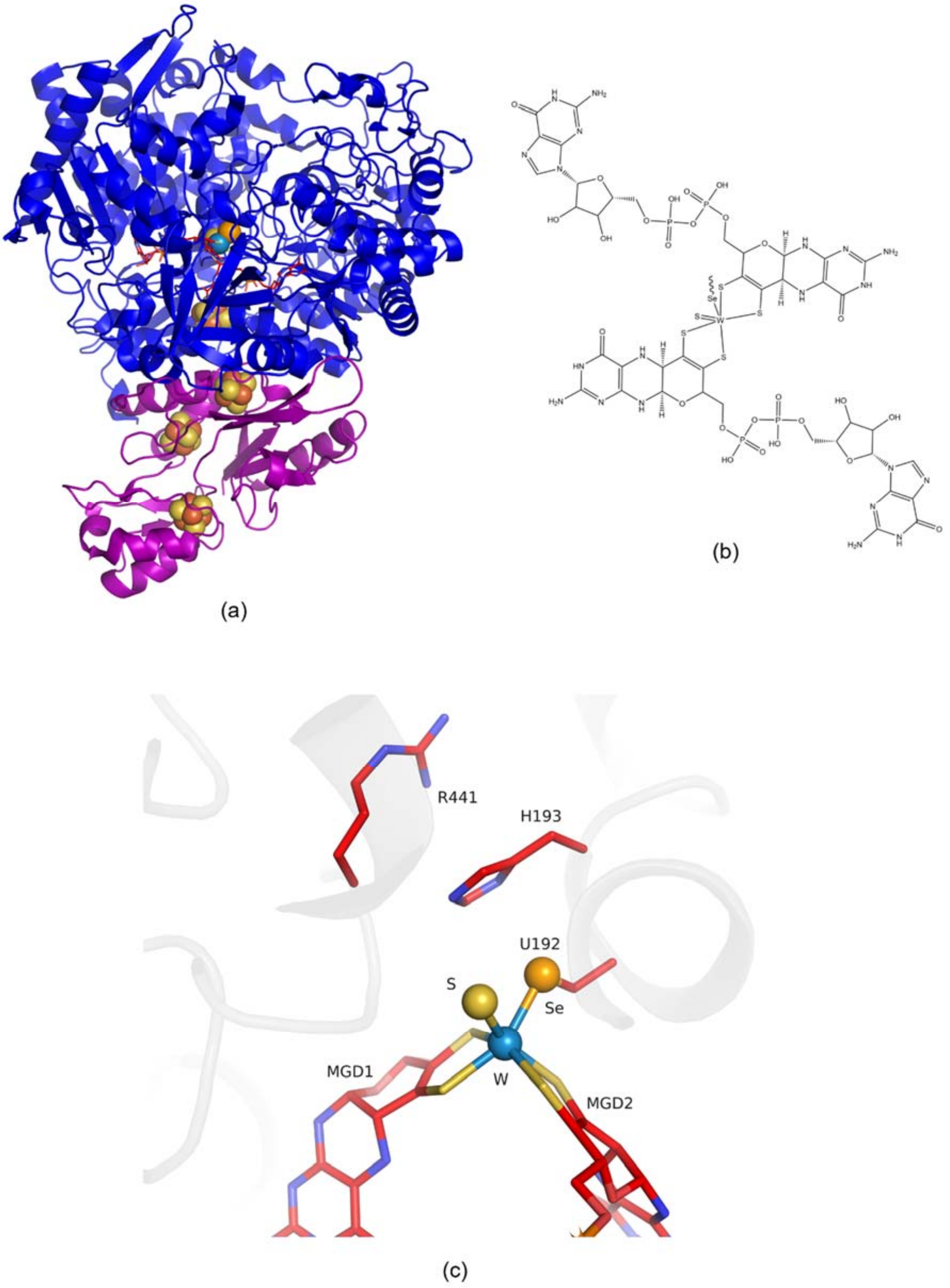


**Figure S1** **(a)** Overall structure of *Dv*FdhAB WT (PDB ID:6SDR), with the α and β subunits in blue and violet, respectively and highlighted the W active site (and its respective 2 MGD co-factors) and the four [4Fe-4S] clusters. **(b)** 2D representation of the W coordination and the two MGD co-factors. **(c)** *Dv*FdhAB WT (PDB ID:6SDR) active site, highlighting the W center and its first (2x MGDs, sulfido and SeCys ligand) and second (H193 and R441) coordination spheres.
